# Evolution of heterotrophy in chrysophytes as reflected by comparative transcriptomics

**DOI:** 10.1093/femsec/fiy039

**Published:** 2018-03-06

**Authors:** Nadine Graupner, Manfred Jensen, Christina Bock, Sabina Marks, Sven Rahmann, Daniela Beisser, Jens Boenigk

**Affiliations:** 1 Biodiversity, Faculty of Biology, University of Duisburg-Essen, Universitätsstr. 5, D-45141 Essen, Germany; 2 Genome Informatics, Institute of Human Genetics, University of Duisburg-Essen, University Hospital Essen, Hufelandstr. 55, D-45147 Essen, Germany; 3 Centre for Water and Environmental Research (ZWU), University of Duisburg-Essen, Universitätsstr. 2, D-45141 Essen, Germany

**Keywords:** *Spumella vulgaris*, *Poteriospumella lacustris*, *Pedospumella encystans*, heterotrophic nanoflagellates (HNF), chrysomonad flagellates, evolutionary ecology, plastid reduction, non-photosynthetic plastids, transcriptome

## Abstract

Shifts in the nutritional mode between phototrophy, mixotrophy and heterotrophy are a widespread phenomenon in the evolution of eukaryotic diversity. The transition between nutritional modes is particularly pronounced in chrysophytes and occurred independently several times through parallel evolution. Thus, chrysophytes provide a unique opportunity for studying the molecular basis of nutritional diversification and of the accompanying pathway reduction and degradation of plastid structures. In order to analyze the succession in switching the nutritional mode from mixotrophy to heterotrophy, we compared the transcriptome of the mixotrophic *Poterioochromonas malhamensis* with the transcriptomes of three obligate heterotrophic species of Ochromonadales. We used the transcriptome of *P. malhamensis* as a reference for plastid reduction in the heterotrophic taxa. The analyzed heterotrophic taxa were in different stages of plastid reduction. We investigated the reduction of several photosynthesis related pathways e.g. the xanthophyll cycle, the mevalonate pathway, the shikimate pathway and the tryptophan biosynthesis as well as the reduction of plastid structures and postulate a presumable succession of pathway reduction and degradation of accompanying structures.

## Teaser

Different pathway repertoirs in heterotrophic chrysophytes indicate the stepwise loss of photosynthesis related pathways in the course of evolution of heterotrophy.

## INTRODUCTION

The changeover in the mode of nutrition from phototrophy to heterotrophy has been surprisingly frequent in the evolution of eukaryotes causing the strikingly scattered distribution of phototrophs and heterotrophs across the eukaryotic tree of life (Cavalier-Smith 2002; Hoef-Emden, Tran and Melkonian 2005; de Koning and Keeling 2006; Krause 2008; de Castro, Gaedke and Boenigk 2009; Keeling 2010; Gile and Slamovits 2014). The independent loss of photosynthetic capability in closely related lineages is particularly pronounced in chrysomonad flagellates (Boenigk *et al*. 2005; Cavalier-Smith and Chao 2006; Grossmann *et al*. 2016): Chrysophyceae possess a plastid, or the remnants of a plastid, originating from secondary endocytobiosis (Delwiche 1999; Keeling 2013). Many taxa are heterotrophic but phylogenetic analyses do not support a basal split into a photo-/mixotrophic and a heterotrophic clade (Andersen *et al*. 1999; Boenigk *et al*. 2005; del Campo and Massana 2011; Grossmann *et al*. 2016). The loss of photosynthesis in many chrysophyte lineages is an excellent example of parallel evolution of heterotrophy from phototrophic or mixotrophic ancestors. Thus, these organisms are the ideal model case for studying various stages of plastid reduction and accompanying changes on pathway level introduced by switching the basic nutritional mode.

The mixotrophic mode of nutrition is widespread amongst several protist lineages (Sanders *et al*. 2001; Caron *et al*. 2016). The ecological relevance of this group is pronounced as mixotrophs can dominate the planktonic community (Sanders 1991; Jones 2000), and some species are common taxa in harmful algal blooms (Anderson, Glibert and Burkholder 2002). The mixotrophic spectrum covers nutritional modes from primarily phototrophic to primarily heterotrophic with all the different gradations being realized. Furthermore, the relative contribution of phototrophy and heterotrophy in distinct taxa may change depending on environmental conditions such as light intensity and the availability of bacteria (Sanders 1991; Jones 2000; Liu *et al*. 2015; Beisser *et al*. 2017b). As shifts in the prevailing mode of nutrition usually require more than 24 h, a switch of nutritional mode within mixotrophs is no subject of a diurnal variation (Sanders, Porter and Caron 1990).

The herein investigated *Poterioochromonas malhamensis* is a model species for mixotrophs with a predominantly heterotrophic mode of nutrition (Caron, Porter and Sanders 1990; Sanders, Porter and Caron 1990; Rottberger *et al*. 2013). Sanders, Porter and Caron (1990) demonstrated that phototrophy contributed less than 7% to the carbon budget of *P. malhamensis*. Furthermore, *P. malhamensis* is dependent on certain vitamins and lacks nitrate-uptake mechanisms (Sanders *et al*. 2001; Terrado *et al*. 2015). Thus, this species may have a reduced genetic repertoire as compared to true phototrophs.

Taxa that shift to an exclusively heterotrophic mode need to ingest carbon and nutrients from the environment. A complete loss of the plastid is postulated only for few lineages or species, in particular for oomycetes, ciliates and some dinoflagellates (Saldarriaga *et al*. 2001; Archibald 2008; Gould, Waller and McFadden 2008; Reyes-Prieto, Moustafa and Bhattacharya 2008; Beakes, Glockling and Sekimoto 2012). In most cases, the loss of photosynthetic capabilities is accompanied by a structural reduction of the plastid but not by a complete loss (Gould, Waller and McFadden 2008). Most of the secondarily heterotrophic lineages still possess remains of their plastids as these are also involved in several metabolic processes other than photosynthesis (de Koning and Keeling 2006). This comprises biosynthesis of biomolecules, specifically of lipids, amino acids, isoprenoids and vitamins. But the plastid located pathway repertoire varies depending on the species (Kamikawa *et al*. 2017). For heterotrophic chrysophytes, Beisser *et al*. (2017) already demonstrated based on transcriptome analyses that these taxa are characterized by a reduced or down-regulated repertoire of genes related to photosynthesis but an increased or up-regulated repertoire of pathways associated with food uptake and motility. The degree of parallel evolution as well as differential trends in the gradual process of structural reductions and pathway degradation in the course of plastid reduction have so far not been comparatively investigated on the molecular level for lineages of heterotrophic chrysophytes in different stages of plastid reduction.

Our research objective is to shed light on the mechanisms behind the pathway reduction and degradation of plastid structures related to the switch from mixotrophy to heterotrophy. The scope of our analysis is to develop a conceptual idea on metabolic changes affiliated with plastid reduction. We therefore focused on a closely related group of organisms i.e. members of the order Ochromonadales rather than the class Chrysophyceae in order to keep interference with phylogenetic signals as low as possible. However, as no exclusive phototrophic member is known for Ochromonadales, we included *Synura* sp. (Synurales) as a reference. The strains used in our study were part of the overarching study of Beisser *et al*. (2017), which presented a first draft of the reduction of photosynthesis. While Beisser *et al*. (2017) focused on the general identification of pathways of central importance for the different modes of nutrition, we extended these investigations by detailed analyses of additional genes and pathways of the biosynthesis of pigments, amino acids, vitamins as well as genes indicative for the presence of distinct plastid structures. Amongst these are genes of the xanthophyll cycle, the mevalonate pathway, the shikimate pathway, the tryptophan biosynthesis as well as glycerolipids. Furthermore, we compared and classified the strains along the range of heterotrophy to develop a model for the gradual process of plastid reduction in chrysophytes.

## MATERIAL AND METHODS

We focused on strains affiliated with the order Ochromonadales, particularly on three colorless strains: *Poteriospumella lacustris* JBM10, *Spumella vulgaris* 199hm and *Pedospumella encystans* JBMS11 and the mixotrophic *Poterioochromonas malhamensis* DS, which we used as reference. Additionally, we use the transcriptome of the phototrophic *Synura* sp. strain LO234KE for the determination of the position of *P. malhamensis* within the mixotrophic spectrum under the utilized culturing conditions. From all other analyses *Synura* sp. was excluded.

### Cultivation of strains and RNA isolation

All strains were grown at 15°C and 75–100 µE at 16:8 hour illumination. For the heterotrophic strains and the mixotrophic strain an inorganic basal medium (Hahn *et al*. 2003) was used. *Spumella vulgaris* and *Pedospumella encystans* were fed with *Listonella pelagia* CB5 (Hahn 1997), whereas the axenic strains of *Poteriospumella lacustris* and *Poterioochromonas malhamensis* were supplemented with 1 g/l of each of nutrient broth, soytone and yeast extract (Hahn *et al*. 2003). For the phototrophic *Synura* sp. strain LO234KE a DY-V medium (Andersen 2007) was used.

All cultures were in the exponential growth phase for several days prior to harvesting via centrifugation (3000 g, 5–10 min, 20°C). RNA isolation was performed under sterile conditions using a TRIzol (Life Technologies, Paisley, Scotland) based protocol (for details see Beisser *et al*. 2017). Quality and quantity of RNA was assessed using the Nanodrop2000 spectrometer (Thermo Fisher Scientific, Darmstadt, Germany) and by the sequencing provider (Eurofins MWG, Ebersberg, Germany).

### Sequencing, assembly and annotation

Transcriptomes were generated in an overarching study of 18 chrysophyte strains published in Beisser *et al*. (2017). Methodological details of sequencing, quality control, assembly strategy and annotation are available from Beisser *et al*. (2017). Poly-A selection, construction of cDNA-libraries and paired-end sequencing on the Illumina HiSeq2000 platform were performed by a commercial service (Eurofins MWG, Ebersberg, Germany). Quality of bases and raw sequence reads was checked using the FastQC software (v0.10.1; Andrews 2015) and preprocessed and trimmed by Cutadapt (v1.3; Martin 2011). The de novo assemblies were carried out using Trinity (release 2013-11-10; Grabherr *et al*. 2011) with default parameters. Gene identification and functional annotation were conducted using the similarity search tool RAPsearch2 (v2.15; Zhao, Tang and Ye 2012) with E-value <10^−5^ against the KEGG database (release 2014-06-23; Kanehisa and Goto 2000). Within the KEGG database genes are associated with orthologous groups and thus assigned to KEGG Orthology (KO) identifiers. In the following, we use the term *gene* for the annotated orthologous gene of the considered transcript.

The raw and assembled sequences are available at the European Nucleotide Archive (ENA) under the accession number PRJEB13662.

### Pathway analysis

KEGG orthologous genes (KOs) assignable to KEGG pathways (Kanehisa and Goto 2000) were considered, except those that match for ‘Human Diseases’. The position of *P. malhamensis* within the mixotrophic spectrum was determined by the number of shared and exclusive KOs between the phototrophic, mixotrophic and the three heterotrophic strains by using the R package VennDiagram (Chen and Boutros 2011). For the analysis of pathway reduction the mixotrophic *P. malhamensis* and the three heterotrophic strains *P. lacustris*, *S. vulgaris* and *P. encystans* were compared based on the presence-absence of orthologous genes and by the completeness of the main reaction steps within pathways (KEGG modules; Kanehisa *et al*. 2017). We focused on pathways related to the mode of nutrition, i.e. photosynthesis, pigments, vitamins and amino acids. Only those pathways that were expressed by the mixotrophic reference strain *P. malhamensis* (indicated by nearly complete main reaction ways of these pathways) were chosen for the analysis of pathway reduction in the heterotrophic strains.

Additionally, the ability to perform *in vivo* photosynthesis was analyzed via measuring the chlorophyll-a fluorescence by using an Aquapen-C AP-C 100 instrument (Photon Systems Instruments, Drasov, Czech Republic). For this purpose, aliquots (10 ml) of a *P. malhamensis* culture were exposed to darkness for at least 30 min before measurements. We used the ‘OJIP’ protocol (measuring phases of the fluorescence transient—O characterize the initial fluorescence (F_0_) and P characterize the maximal level) for measuring photosystem II integrity and we measured light curves (dependent on enzymatic ‘dark’ reactions) (Strasser, Srivastava and Tsimilli-Michael 2000; Boisvert, Joly and Carpentier 2006; Thwe and Kasemsap 2014). Both measurements were recorded 10 times, clearly indicating photosynthetic activity of this strain.

## RESULTS AND DISCUSSION

### Transcriptome sizes and quality of assemblies

Sequencing resulted in 13.3–19.4 million read pairs, which could be assembled into 26330–58003 transcripts (Table 1). The high quality of the assemblies was reflected by a high percentage of remappable reads to the assembled transcripts (92% on average) and an average N50 value of 1178 nucleotides, whereby the assembly quality of the phototrophic strain, which was only used for the classification of the nutritional position of the mixotrophic *P. malhamensis*, was not as good as the assemblies of the other strains (N50: 492, remapped reads: 72%). Furthermore, the completeness of KEGG modules (at most one gene may be missing), i.e. of the main reaction steps in KEGG pathways, of pathways related to the primary metabolism such as glycolysis, citrate cycle and nucleotide biosynthesis denoted a sufficient sequencing depth in all samples.

**Table 1.** Overview statistics of the phototrophic *Synura* sp. (LO234KE), the mixotrophic *Poterioochromonas malhamensis* (DS) and the heterotrophic strains *Poteriospumella lacustris* (JBM10), *Spumella vulgaris* (199hm) and *Pedospumella encystans* (JBMS11) including transcriptome size, assembly quality and annotation.

	*Synura sp. (LO234KE)*	*P. malhamensis* (DS)	*P. lacustris* (JBM10)	*S. vulgaris* (199hm)	*P. encystans* (JBMS11)
No. read pairs (million)	13.3	18.8	19.4	13.9	14.2
Reads after quality control (%)	43.2	94.3	91.7	92.4	93.0
No. transcripts	43120	39537	26330	58003	40532
N50	492	1405	1246	983	1077
Remapped reads (%)	72.2	95.1	97.0	89.5	86.1
Estimated no. of protein-coding genes	29955	30189	20515	38883	28497
No. KEGG orthologs assignable to pathways	1786	1599	1389	1635	1591
No. assigned KEGG pathways	255	247	246	259	257

### *P. malhamensis*—relative importance of heterotrophy and phototrophy

**Figure 1.**
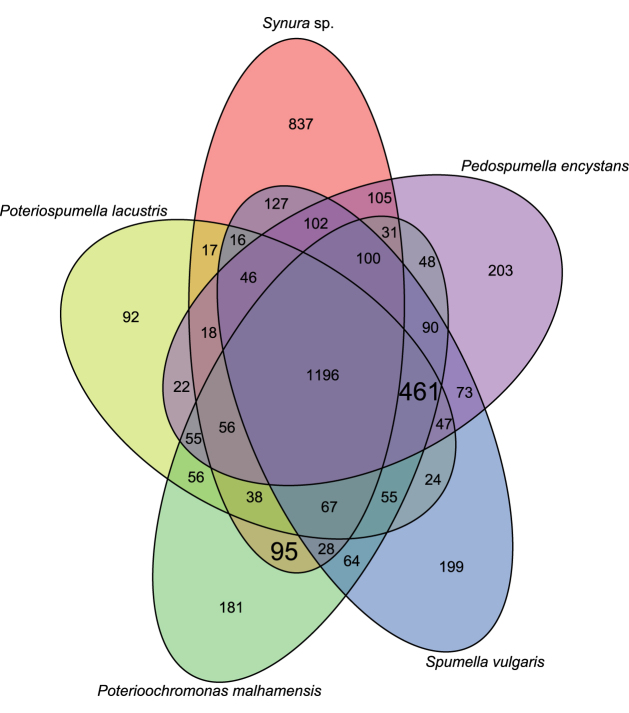
Venn diagram of KEGG orthologous genes (KOs) of the phototrophic *Synura* sp., the mixotrophic *Poterioochromonas malhamensis* as well as the three heterotrophic species *Poteriospumella lacustris*, *Spumella vulgaris* and *Pedospumella encystans*. The numbers in bigger font are the number of exclusively shared KOs between the phototrophic *Synura* sp. and the mixotrophic *P. malhamensis* and the number of KOs exclusively shared between the mixotrophic *P. malhamensis* and the three heterotrophs.

The comparison of the KEGG orthologous genes separated the phototrophic species *Synura* sp. from all other species included in the analysis, i.e. the mixotrophic *Poterioochromonas malhamensis* and the three heterotrophic species. *P. malhamensis* shared a high number of genes with all heterotrophic species (Fig. 1). Beisser *et al*. (2017) demonstrated that chrysophycean strains with the same nutritional strategy express a shared gene set independent of their phylogeny. The observed separation of strains is therefore presumably largely due to the predominantly heterotrophic mode of nutrition of *P. malhamensis*. A certain bias due to the phylogenetic relation of the investigated strains cannot be ruled out as *Synura* sp. is a member of the order Synurales, whereas all other investigated strains are members of the order Ochromonadales and thus more closely related. However, the idea that the mixotrophic *P. malhamensis* predominantly expressed genes associated with heterotrophy as suggested by Beisser *et al*. (2017) was supported by *in vivo* control experiments based on chlorophyll fluorescence emission (Aquapen-C). These experiments proofed functional light and dark reactions (typical course of light curves) of photosynthesis in *P. malhamensis* but the maximal photon use efficiency analyzed in OJIP curves was low (≤0.33) as compared to phototrophic *Synura* sp. or Chlorophyta (≥0.6). The reason for this inefficient PSII is unclear but matches the predominantly heterotrophic type of nutrition. The primarily heterotrophic metabolism of *P. malhamensis* is in accordance with the findings of previous studies (e.g. Caron, Porter and Sanders 1990; Sanders, Porter and Caron 1990; Rottberger *et al*. 2013; Beisser *et al*. 2017). The transcribed genetic repertoire of *P. malhamensis* may thus represent a minimal photosynthetic repertoire close to the border of exclusive heterotrophy.

### Genes and pathways indicating phototrophy or heterotrophy

**Figure 2.**
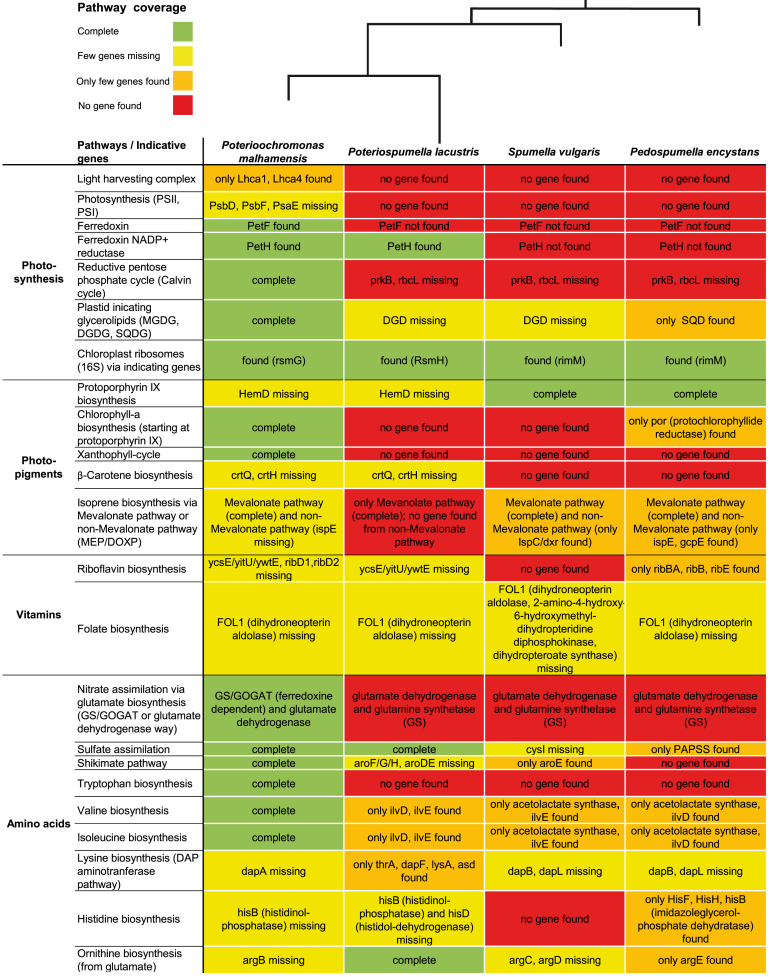
Pathway reduction linked to nutritional strategies of the mixotrophic *Poterioochromonas malhamensis* (DS), and the three heterotrophic strains *Poteriospumella lacustris* (JBM10), *Spumella vulgaris* (199hm) and *Pedospumella encystans* (JBMS11). The illustrated phylogenetic relation between the investigated strains is based on the SSU phylogeny of Grossmann *et al*. (2016) that covers all known linages of Chrysophyceae.

Nearly all pathways and genes that indicate structures or functions typical for phototrophy were expressed in the mixotrophic *P. malhamensis* but not expressed at the time of sampling or reduced in the heterotrophic strains (Fig. 2). Expression profiles indicated different stages of reduction as some pathways or genes were not expressed or reduced in all heterotrophic strains, whereas other pathways or genes were still present in the transcriptomes of at least one heterotrophic strain but missing in others.

#### Genes indicating an intact photosynthesis machinery and the presence of a plastid

The genes present in the transcriptome of the mixotrophic *Poterioochromonas malhamensis* indicated an intact photosynthesis machinery (Fig. 2). The expressed genes included several genes coding for the genes Lhca1 and Lhca4 of the light harvesting complex, for the photosystems PSI and PSII, for ferredoxin and for ferredoxin NADP^+^ reductase, as well as for all genes of the reductive pentose phosphate cycle (Calvin cycle). The complete process of photosynthesis, which begins with light absorption by antenna proteins and ending with stable carbon products (Blankenship 2008), was shown by these pathways. We also found genes coding for the enzymes digalactosyldiacylglycerol synthase (DGD), monogalactosyldiacylglycerol synthase (MGD) as well as UDP-sulfoquinovose synthase and sulfoquinovosyltransferase (Fig. 2). The latter genes catalyze the synthesis of the glycerolipids digalactosyldiacylglycerol (DGDG), monogalactosyldiacylglycerol (MGDG) and sulfoquinovosyldiacylglycerol (SQDG), which indicated the structural presence of a plastid. MGDG and DGDG taken together account for 75% of the lipids in the thylakoid membrane (Gounaris and Barber 1983; Dörmann and Benning^2002^; Hölz *et al*. 2009) and they also build the plastid membrane (Gounaris and Barber 1983; Petroutso *et al*. 2014). Genes coding for these lipids were therefore used to detect the primary photosynthetic plastids (Botté and Maréchal 2014) or plastid remnants (Leblond, Dodson and Dahme 2013). The presence of a plastid was further supported by the expression of ribosomal genes of the chloroplasts (Fig. 2).

In contrast to *P. malhamensis*, most of the above genes were not expressed in the heterotrophic strains. In particular, the heterotrophic species did not express any of the genes directly associated with photosynthesis, i.e. genes coding for the light harvesting complex, PSI and PSII, ferredoxin, and the rbcL gene (coding for RuBisCO) of the reductive pentose phosphate cycle (Fig. 2).

However, whereas genes indicative for photosynthesis were not found in the expression profiles of the heterotrophic strains, genes indicative for the presence of a plastid were expressed by the heterotrophs. In particular, genes coding for DGD, MGD and SQD were found in the heterotrophic strains but the number of identified genes differed between the strains. We found MGD and SQD in *P. lacustris* and *S. vulgaris* whereas only SQD was detectable in *P. encystans*. We also found genes coding for plastidal ribosomal proteins in all heterotrophic species. Interestingly we found the gene PetH coding for the ferredoxin NADP^+^ reductase in the transcriptome of *P. lacustris* but not in the transcriptomes of any other heterotrophic species (Fig. 2).

#### Genes coding for photo-pigments

Pigments are an obligatory requirement for photosynthesis as they absorb light and convert it into energy (Blankenship 2008). Most genes for the biosynthesis of photosynthetic pigments including protoporphyrin IX (precursor-component of chlorophyll and heme), chlorophyll-a and β-carotene as well as all genes of the xanthophyll cycle were found in the transcriptome of *P. malhamensis* (Fig. 2). Different chlorophylls and carotenoids together with proteins form the light harvesting complexes of the photosynthetic membranes (Siefermann-Harms 1987). An additional function of carotenoids is the photo-protection (Siefermann-Harms 1987; Blankenship 2008). The carotenoid violaxanthin is converted to zeaxanthin under excess light conditions for photo-protection and vice versa under limiting light conditions (Blankenship 2008). The expression of the pigment biosynthesis genes was in different stages of reduction in the heterotrophs. Basically, the protoporphyrin biosynthesis was nearly completely expressed by the heterotrophic strains but no heterotrophic strain expressed genes for the chlorophyll-a biosynthesis (except protochlorophyllide reductase in *P. encystans*). Furthermore, no gene for the xanthophyll cycle was expressed by the heterotrophic strains. β-carotene biosynthesis was nearly completely expressed in *P. lacustris* but strongly down-regulated or not expressed in the other species (Fig. 2).

In the context of pigment biosynthesis, we also investigated the expression of genes coding for isoprene biosynthesis as the terpenoid backbone is amongst others a precursor of carotenoids. In *P. malhamensis* two pathways of biosynthesis were expressed: the non-mevalonate pathway (MEP/DOXP) that is typically located in the plastids or in bacteria and produces the precursor of beta-carotene, lutein, prenyl chains of chlorophylls and plastoquinone (Lichtenthaler *et al*. 1997) as well as the mevalonate pathway that is located in the cytosol and produces the precursor of sterol (Lichtenthaler *et al*. 1997). In contrast to *P. malhamensis*, the heterotrophic strains expressed only the mevalonate pathway. The non-mevalonate pathway was not expressed in *P. lacustris* and we detected only the expression of distinct genes in the transcriptomes of *S. vulgaris* and *P. encystans* (Fig. 2). These genes (IspC/dxr, IspE and GcpE) are also expressed by the colpodellid *Voromonas pontica*, which has a reduced non-photosynthetic plastid (Gile and Slamovits 2014). Additionally, in several dinoflagellates and apicomplexans with cryptic plastids were single genes e.g. the IspC/dxr gene found (Sanchez-Puerta *et al*. 2007; Slamovits and Keeling^2008^).

#### Genes coding for vitamin and amino acid biosynthesis

It is known that algae have the capability to synthesize vitamins but several algae also require certain vitamins. Amongst these frequently required vitamins are vitamin B12 (cobalamin), vitamin B1 (thiamine) and vitamin B7 (biotin) (Croft, Warren and Smith^2006^; Petroutsos *et al*. 2014). We identified only few genes indicating vitamin synthesis in the mixotrophic *P. malhamensis*. Amongst these were genes coding for riboflavin (vitamin B2) and folate (vitamin B9 or folacin) (Fig. 2). Liu *et al*. (2016) found all genes of the riboflavin pathway in the transcriptomes of *Dinobryon* sp. and *Ochromonas* sp., two mixotrophic chrysophytes. While several genes for the biosynthesis of folate were expressed in all heterotrophs, none were expressed for the riboflavin biosynthesis in *S. vulgaris* and only few in *P. encystans*. Taken together, these may indicate that in general mixotrophic chrysophytes are able to synthesize riboflavin (vitamin B2).

The biosynthesis of amino acids is dependent on the supply of nitrogen and, in case of few sulfur containing amino acids, also on sulfur (Graham, Wilcox and Graham 2009). Therefore, we investigated the nitrogen and sulfur assimilation. During nitrogen assimilation, ammonium (direct uptake of ammonium or uptake of nitrate which is reduced to ammonium via nitrite) is used for the synthesis of glutamate, which is the starting point for the synthesis of several other amino acids. The synthesis of glutamate can be performed by two ways - direct via the glutamate dehydrogenase (Miflin and Lea^1976^; de Castro, Gaedke and Boenigk 2009) or via a two step-reaction that was firstly described by bacteria, called GS/GOGAT, where the glutamine synthetase catalyzes the reaction to glutamine and subsequently the glutamate synthase catalyzes the reaction to glutamate (Miflin and Lea 1976; Miflin and Habash 2002; Röttgers 2002). Both pathways, the GS/GOGAT pathway and the glutamate dehydrogenase pathway were expressed by *P. malhamensis* (Fig. 2). During sulfur assimilation, sulfate is reduced to sulfide via sulfite and subsequently sulfide is used for the biosynthesis of cysteine, which is the educt for the synthesis of methionine, another important sulfur containing amino acid (Droux 2004). All genes related to sulfate assimilation were found in the transcriptome of *P. malhamensis*. Furthermore, the precursor pathway for aromatic amino acids, the shikimate pathway (Herrmann and Weaver^1999^), was also completely expressed by *P. malhamensis*.

In the heterotrophs, we found genes coding for the glutamate dehydrogenase and for the glutamine synthethase but not for the glutamate synthase. Thus, the heterotrophs presumably only perform the glutamate dehydrogenase way. The genes for the sulfate assimilation were expressed by *P. lacustris* and *S. vulgaris*, but downregulated or missing in *P. encystans* except for one gene of this pathway. Furthermore, we found indications for a complete transcription of the shikimate pathway only in one heterotrophic strain (*P. lacustris*), whereas the other heterotrophs did not express these genes or only few of them (Fig. 2).

The majority of machinery for the biosynthesis of amino acids is located in the plastid (Lancien, Lea and Azevedo 2006). Several amino acids were synthesized by all investigated strains but some pathways for the biosynthesis of amino acids were found exclusively in the mixotrophic *P. malhamensis*. The tryptophan biosynthesis was not expressed by the heterotrophs and in the biosynthesis pathways for valine and isoleucine genes were only expressed scattered (Fig. 2).

### Model for the succession of pathway and structural reduction of plastids in heterotrophs

**Figure 3.**
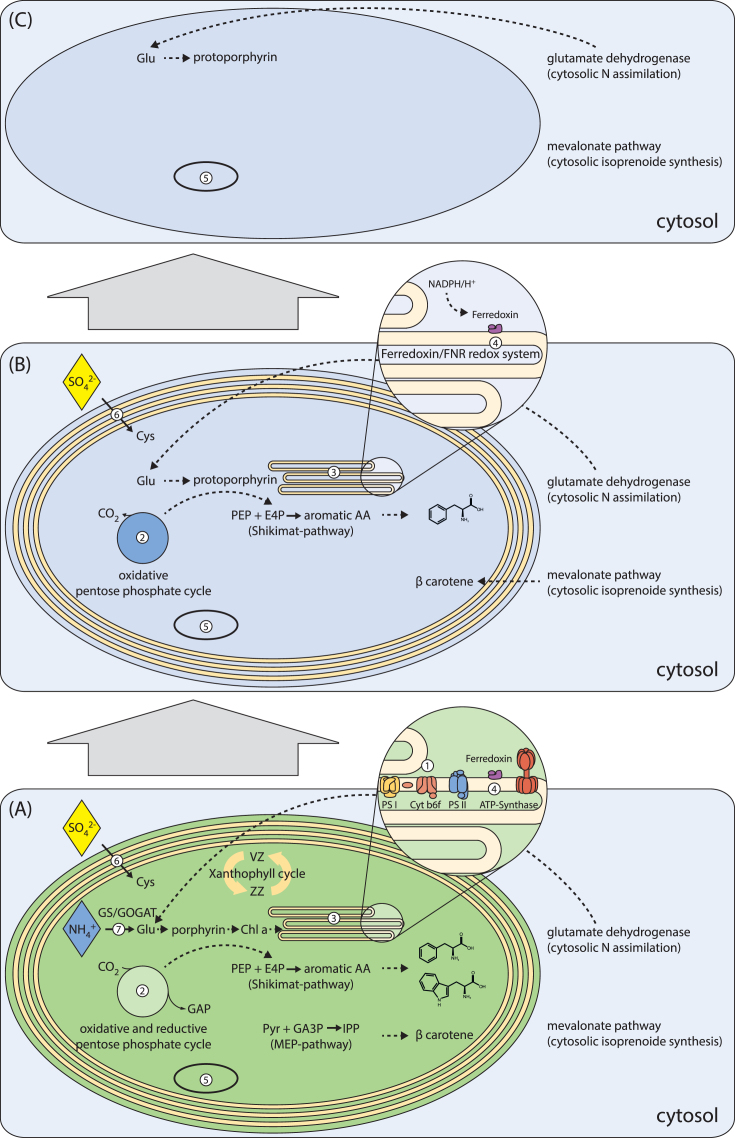
**(A–C)** Model for plastid reduction. (**A**) Photosynthetic plastid with its (5) plastome and an intact photosynthesis machinery including the structural requirements for it: (1) light reaction, (2) oxidative & reductive pentose phosphate cycle, (3) thylakoids with (4) ferredoxin as well as additional plastid located pathways are expressed: (6) sulfateassimilation, (7) nitrogenassimilation via the GS/GOGAT way and subsequent protoporphyrin and chlorophyll-a synthesis, the synthesis of β-carotene via the isoprenoidsynthesis in the MEP pathway, the xanthophyll-cycle and the synthesis of aromatic amino acids via the shikimate pathway. The nitrogenassimilation has a cytosolic alternative, the glutamate dehydrogenase way and the isoprenoidsynthesis via the MEP pathway has as cytosolic alternative the mevalonate pathway. (**B**) Plastid in an early stage of reduction with its (5) plastome. Photosynthesis is reduced so that only the (2) oxidative and not the reductive pentose phosphate cycle is expressed, (3) thylakoids are present with a (4) ferredoxin/FNR redox system. Among the additional pathways that are expressed in the plastid are the (6) sulfateassimilation and the shikimate pathway for the synthesis of aromatic amino acids, whereby tryptophan is no longer synthesized. The β-carotene synthesis is now expressed via the cytosolic mevalonate pathway, the synthesis of chlorophyll-a and the plastid located nitrogenassimilation are no longer express like the xanthophyll-cycle. However, the protoporphyrin synthesis via the cytosolic nitrogen assimilation is expressed in the plastid. (**C**) Plastid in an advanced stage of reduction with its (5) plastome. The (3) thylakoids are lost and all additional biosynthesis except the biosynthesis of protoporphyrin are no longer expressed.

The loss of the photosynthetic capability and thus the shift to the heterotrophic mode of nutrition of the herein investigated species occurs presumably independently from each other and thus is an example of parallel evolution. Based on the investigations of the expressed genetic repertoire in these heterotrophs, which are in different stages of plastid reduction, we postulate a two-step model for the gradual reduction of pathways and structures in heterotrophic chrysophytes (Fig. 3. A–C)

#### Starting situation of plastid reduction

We used the mixotrophic chrysophyte *P. malhamensis* that is already far on the heterotrophic side of the nutritional spectrum of mixotrophy but still able to perform photosynthesis (Caron, Porter and Sanders 1990; Rottberger *et al*. 2013) as reference strain for basal photosynthetic activity. The structural presence of a plastid is confirmed in *P. malhamensis* by the transcription of genes coding for lipids of the plastid membrane as well as for plastid-located pathways. Furthermore, genes and pathways of the photosynthesis as well as genes for the biosynthesis of photo-pigments were found in the transcriptome of *P. malhamensis* (Fig. 3A). Taken together the expressed genes indicate an intact photosynthesis machinery.

Inorganic nitrogen and sulfur uptake mechanisms are an essential prerequisite for amino acid synthesis in phototrophic organisms. Also from mixotrophs the uptake of nitrate or ammonium as inorganic nitrogen source is known (Caron, Porter and Sanders 1990; Lewitus *et al*. 1999; Liu *et al*. 2016). Even though *P. malhamensis* is already rather heterotrophic, we identified genes coding for both assimilation pathways (nitrogen and sulfur), the precursor pathway of aromatic amino acids and several amino acids (Fig. 3A). Mixotrophic organisms may, despite a certain nutrient uptake via phagocytosis, still experience nutrient limitations and the expression of these pathways therefore seems sensible. The expression of biosynthesis pathways for amino acids in *P. malhamensis* underlines the high degree of nutritional autonomy in comparison to obligatory heterotrophs. Nevertheless, some biomolecules, in particular several vitamins, can probably not be synthesized by *P. malhamensis* as this is known for other chrysophytes as well (Droop 1954; Heinrich 1955; Liu *et al*. 2016). Summarizing, our data confirm that *P. malhamensis* can synthesize most biomolecules to ensure survival without the ingestion and digestion of food, which is in accordance with previous studies (e.g. Caron, Porter and Sanders 1990; Rottberger *et al*. 2013).

In contrast, the three investigated heterotrophic species are in different stages of plastid reduction and, concomitantly, of pathway reduction (Fig. 3B and C). *Poteriospumella lacustris* has a reduced or not expressed inventory of genes associated with photosynthesis but several pathways related to photosynthesis are still completely or almost completely expressed. Thus, *P. lacustris* is a heterotrophic chrysophyte that has presumably shifted the nutritional mode more recently, and it therefore serves as a model for an early stage in plastid reduction. In *S. vulgaris* and *P. encystans* plastid reduction seems to be more advanced and photosynthesis-related pathways are largely missing in the transcriptome. These taxa therefore serve as reference organisms for advanced stages in the plastid reduction.

#### Early stage of plastid reduction

We postulate that the first step of plastid reduction is characterized by the absence of genes directly related to photosynthesis. In particular, genes coding for the light harvesting complex, PS I and PS II, the reductive pentose phosphate cycle (but the oxidative pentose phosphate cycle is still present) and biosynthesis of chlorophyll-a are reduced or not expressed (Fig. 3B). The early reduction of the photosystems is in accordance with the findings of Hadariová *et al*. (2017), who developed a model for the reduction of plastid genomes based on a literature review including non-photosynthetic plants, algae and protists. In their model, *psa* and *psb* genes that are located in the plastid genome are the second step in plastid genome reduction (after the reduction of *ndh* genes). Beyond the reduction of photosynthesis, additionally pathways that are more indirectly linked to photosynthesis are affected: For instance, photo-protection that is required in photosynthetic organisms is reduced (in this case, the xanthophyll-cycle). Furthermore, several plastid-located pathways that have an alternative variant in the cytosol are not expressed or missing (while the cytosolic way still remains) indicating that the importance of the plastid is not only reduced with respect to photosynthesis but also to other pathways (Fig. 3B). Particularly worth mentioning is the plastid located non-mevalonate pathway as several cryptic-plastid-bearing species of colpodellids, apicomplexians and dinoflagellates can express this pathway (Sanchez-Puerta *et al*. 2007; Gile and Slamovits^2014^; Janouškov *et al*. 2017). For three dinoflagellate species, evidence exists that they lack the cytosolic variant (Janouškovec *et al*. 2017). In our study, however, we could identify the cytosolic way in all investigated chrysophytes. Therefore, the single genes found in two of the three heterotrophic species most probable indicate a reduction with subsequent loss of this pathway like in the non-photosynthetic diatom *Nitzschia* sp. (Kamikawa *et al*. 2017). Likely, the organisms developed during the course of plastid reduction an increasing independence from plastid-located metabolic pathways. Furthermore, some pathways for amino acids synthesis, e.g. for the aromatic amino acid tryptophan, are reduced or not expressed also indicating the increasing contribution of heterotrophy to the acquisition of biomolecules. But the biosynthesis of aromatic amino acids, e.g. phenylalanine is still possible via the shikimate pathway requiring erythrose-4-phoshpat from the oxidative pentose phosphate cycle (Fig. 3B).

Concomitantly to this metabolic reduction, we assert a first structural reduction of the plastids, as DGD, i.e. a gene that synthesizes one of two main lipids of the thylakoid/plastid membrane, was not found in the transcriptome. However, MGD, i.e. the enzyme synthesizing the other main lipid of the thylakoid/plastid membranes, as well as one more enzyme for thylakoid/plastid membrane lipids, i.e. SQD, and genes coding for the ribosomes of the plastid were still found in the transcriptome and thus indicate the structural presence of the plastid. Furthermore, we identified pathways that require the plastid. For instance, we found evidence in *P. lacustris* for the biosynthesis of beta-carotenoids. Furthermore, the presence of PetH, which is coding for the ferredoxin NADP^+^ reductase, implies that reductive synthesis are presumably still located in the plastid. This enzyme typically reduces the reduction equivalents NADP^+^ to NADPH at the end of the PSI reactions. We presume a ferredoxin-FNR (ferredoxin-reductase) redox system similar to those identified in the apicomplexian parasites by Vollmer *et al*. (2001) and Pandini *et al*. (2002), which transfers electrons in the opposite direction, i.e. from NADPH to ferredoxin (Fig. 3B). This kind of redox system was also identified in non-photosynthetic dinoflagellates (Janouškovec *et al*. 2017). Even though the extent of the structural reduction of plastids cannot be precisely predicted from transcriptome data, the postulated pathway reduction is backed up by electron microscopic evidence of plastids and thylakoids reported by Grossmann *et al*. (2016). The thylakoids were identified in another heterotrophic strain, not the herein investigated *P. lacustris*, but based on the electron microscopic results the presence of thylakoids in *P. lacustris* cannot be excluded. Consequently, we assume that thylakoids are still present at the early stage of heterotrophy (Fig. 3B).

A similar extent of pathway reduction and structural reduction was identified in the non-photosynthetic diatom of the genus *Nitzschia* (Kamikawa *et al*. 2015; Kamikawa *et al*. 2015b; Kamikawa *et al*. 2017). The loss of photosynthesis in this diatom is presumed to occur relative recently as thylakoids are still present in the plastid, proven by electron microscopic identification (Kamikawa *et al*. 2015b) and plastome encoded genes for the ATP complex, which is located in the thylakoid membrane (Kamik *et al*. 2015).

#### Advanced stage of plastid reduction

For advanced stages of plastid reduction, we assume that reductive synthesis pathways, requiring NADPH for the reduction of ferredoxin, as well as beta-carotene biosynthesis and riboflavin biosynthesis, are also reduced (Fig. 3C). This reduction is accompanied by an advanced reduction of the plastid indicated by the loss of another enzyme synthesizing the thylakoid/plastid membrane lipid, MGDG. This reduction presumably affects the thylakoids (Fig. 3C). Beyond the reduction of the plastid, the organisms increasingly rely on the phagotrophic uptake of biomolecules. Pathways for the synthesis of amino acids are absent or not expressed. In particular, the sulfate assimilation as well as the shikimate pathway is missing (Fig. 3C). Thus, sulfate containing amino acids as well as aromatic amino acids cannot be synthesized but need to be taken up with the food. The lack of amino acid biosynthesis pathways, which is one characteristic feature of the advanced stage of plastid reduction, is known for species with strongly reduced plastids like the apicomplexans e.g. *Plasmodium falciparum* and *Toxoplasma gondii* (Fleige, Limenitakis and Soldati-Favre 2010; Lim and McFadden 2010; Sheiner, Vaidya and McFadden 2013; van Dooren and Striepen 2013). However, not all pathways are reduced in the course of plastid reduction. The protoporphyrin biosynthesis is a pathway that remains active in the plastid independently from the stage of plastid reduction (Fig 3. A–C)

## CONCLUSIONS

We demonstrated based on comparative transcriptomics that the switch from mixotrophy to heterotrophy and of plastid reduction is a gradual process. Our study revealed the extent of both the genetic and the structural differentiation of chrysophytes during the changeover from mixotrophy to heterotrophy. According to expectations, genes belonging to photosynthesis only played a major role in the mixotrop *Poterioochromonas malhamensis*. As *P. malhamensis* behaves nearly heterotrophically we assume that the transcribed photosynthetic repertoire is close to the minimal genetic repertoire required for an intact photosynthesis. The pathway reduction in the heterotrophs underline an increasing need for ingestion and digestion of prey and nutrients for the acquisition of biomolecules. The reduction of photosynthesis and of related pathways in heterotrophic chrysophytes presumably is accompanied by a structural reduction, i.e. a reduction of the thylakoids in an advanced stage of plastid reduction. Our results provide a conceptual basis for the gradual evolution of nutritional differentiation in eukaryotes—in particular stramenopiles.

We would like to emphasize that missing genes in transcriptome data do not necessarily indicate the reduction of genes. However, transcriptomic data are a good first proxy in particular when considering complete pathways and they provide a sufficient basis for the presented hypotheses on metabolic pathway reductions—irrespective on whether the basis for this reduction is gene loss, mutation or down-regulation. Future genomic studies, a necessary next step, may however prove the actual stage of gene losses.
